# Genomic deletion of *CNGB3* is identical by descent in multiple canine breeds and causes achromatopsia

**DOI:** 10.1186/1471-2156-14-27

**Published:** 2013-04-20

**Authors:** Connie Y Yeh, Orly Goldstein, Anna V Kukekova, Debbie Holley, Amy M Knollinger, Heather J Huson, Susan E Pearce-Kelling, Gregory M Acland, András M Komáromy

**Affiliations:** 1 Department of Clinical Studies, School of Veterinary Medicine, University of Pennsylvania, Philadelphia, PA 19104, USA; 2 Department of Small Animal Clinical Sciences, College of Veterinary Medicine, Michigan State University, East Lansing, MI 48824, USA; 3 James A. Baker Institute for Animal Health, College of Veterinary Medicine, Cornell University, Ithaca, NY 14853, USA; 4 Department of Animal Sciences, College of Agricultural, Consumer and Environmental Sciences, The University of Illinois at Urbana-Champaign, Urbana, IL 61801, USA; 5 Willow Creek Pet Center, Cottonwood Heights, UT 84093, USA; 6 Eye Care for Animals, Salt Lake City, UT 84106, USA; 7 Bovine Functional Genomics Laboratory, Agricultural Research Services, United States Department of Agriculture, Beltsville, MD 20705, USA; 8 OptiGen, LLC, Ithaca, NY 14850, USA

**Keywords:** Achromatopsia, Alaskan malamute, Alaskan sled dog, Australian shepherd, Cone degeneration, *CNGB3*, Day-blindness, Identical by descent, Siberian husky

## Abstract

**Background:**

Achromatopsia is an autosomal recessive disease characterized by the loss of cone photoreceptor function that results in day-blindness, total colorblindness, and decreased central visual acuity. The most common causes for the disease are mutations in the *CNGB3* gene, coding for the *beta* subunit of the cyclic nucleotide-gated channels in cones. *CNGB3*-achromatopsia, or cone degeneration (cd), is also known to occur in two canine breeds, the Alaskan malamute (AM) and the German shorthaired pointer.

**Results:**

Here we report an in-depth characterization of the achromatopsia phenotype in a new canine breed, the miniature Australian shepherd (MAS). Genotyping revealed that the dog was homozygous for a complete genomic deletion of the *CNGB3* gene, as has been previously observed in the AM. Identical breakpoints on chromosome 29 were identified in both the affected AM and MAS with a resulting deletion of 404,820 bp. Pooled DNA samples of unrelated purebred Australian shepherd, MAS, Siberian husky, Samoyed and Alaskan sled dogs were screened for the presence of the affected allele; one Siberian husky and three Alaskan sled dogs were identified as carriers. The affected chromosomes from the AM, MAS, and Siberian husky were genotyped for 147 SNPs in a 3.93 Mb interval within the *cd* locus. An identical shared affected haplotype, 0.5 Mb long, was observed in all three breeds and defined the minimal linkage disequilibrium (LD) across breeds. This supports the idea that the mutated allele was identical by descent (IBD).

**Conclusion:**

We report the occurrence of *CNGB3*-achromatopsia in a new canine breed, the MAS. The *CNGB3*-deletion allele previously described in the AM was also observed in a homozygous state in the affected MAS, as well as in a heterozygous carrier state in a Siberian husky and Alaskan sled dogs. All affected alleles were shown to be IBD, strongly suggesting an affected founder effect. Since the MAS is not known to be genetically related to the AM, other breeds may potentially carry the same cd-allele and be affected by achromatopsia.

## Background

Congenital achromatopsia, also called rod monochromacy and day-blindness, is a rare autosomal recessive condition that results in complete loss of cone photoreceptor function, while the rod photoreceptors remain intact [1]. The disease is characterized by decreased visual acuity, photophobia, nystagmus, and complete colorblindness [1]. Thus far, mutations in five genes have been identified to cause achromatopsia; they encode key components of the cone phototransduction cascade: the *alpha* (*PDE6C*) and *gamma* (*PDE6H*) subunits of cone cyclic guanosine monophosphate (cGMP) phosphodiesterase [2,3], the *alpha* subunit of cone transducin (*GNAT2*) [4,5], as well as the *alpha* (*CNGA3*) and *beta* (*CNGB3*) subunits of cone cyclic-nucleotide gated channel [6-8]. In the majority of patients, achromatopsia is a channelopathy and caused by mutations in either the *CNGA3* or *CNGB3* gene, with *CNGB3* being affected most commonly [9-12].

Achromatopsia is naturally occurring in two canine breeds, where the condition is also referred to as cone degeneration (cd). In the Alaskan malamute (AM), it is caused by a genomic deletion of the entire *CNGB3* gene, while in the German shorthaired pointer it results from a missense mutation in exon 6 [13]. Both of these genetic defects are functional null mutations, and the phenotypic manifestations resemble those observed in human patients [14]. The genomic deletion in cd-affected AMs that includes the *CNGB3* gene was not fully characterized. The length of the deletion and the inclusion of other adjacent genes were not identified, which resulted in an inability to identify carrier dogs [13].

The clinical signs of canine achromatopsia, predominantly day-blindness, typically manifest by 8–12 weeks of age when retinal development is completed in dogs [15-18]. Cones develop normally but once they are no longer functional, their inner and outer segments gradually deteriorate, followed by a slow loss of cones throughout the animal’s lifetime [16,19]. The loss of cone function can be confirmed by electroretinography [14,15]. However, affected dogs remain ophthalmoscopically normal.

The main formation of dog breeds took place in the last 200 years. Some breeds have evolved from dogs with unknown ancestry and have been maintained as closed lines, while others were formed by cross-breeding of existing breeds. The relationship among dog breeds at the molecular level has been established [20,21]. It is not surprising that closely related breeds often share diseases caused by the same identical by descent (IBD) allele. It is less expected that a disease could be caused by an IBD mutation in not closely related breeds. Identification of common disease mutations among breeds that are not closely related indicates that large scale genetic screening of unrelated breeds can provide unexpected information about disease-causing alleles segregating in individual breeds. In the present study, we report in-depth the identical phenotype and genotype of AM-achromatopsia in a new, unrelated canine breed, the miniature Australian shepherd (MAS). We also found the same cd-allele in a Siberian husky and Alaskan sled dogs in a heterozygous state. We identified the breakpoints and determined the length of the genomic *CNGB3*-deletion to be 404,820 bp, involving two other genes. All the cd-alleles found in the different breeds were shown to be IBD, strongly suggesting an affected founder effect.

## Results

### Determining the deletion breakpoints of the *CNGB3* mutation in the Alaskan malamute (AM) breed and establishing a diagnostic test

**Figure 1:**
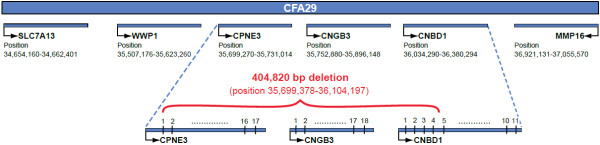
**Schematic presentation of the *cd *locus and the *CNGB3 *deletion.** The initial screening confirmed the presence of four genes within the disease locus: *SLC7A13*, *WWP1*, *CNBD1*, and *MMP16*. Fine mapping identified a 404,820 bp deletion at position 35,699,378-36,104,197 that included the distal end of *CPNE3*-exon 1 and continued to include the rest of the gene, the complete *CNGB3* gene, and the first four exons of *CNBD1*, to end in the middle of intron 4. Numbered black lines represent exons. Figure is not drawn to scale.

The exact size of the genomic deletion on CFA29 that includes the *CNGB3* gene in cd-affected AMs has not yet been defined. BAC clone analysis and FISH hybridization suggested that the deletion should be between 150 kb and 1 Mb [13]. To identify the breakpoints of the deletion, we sought to genotype an AM-derived cd-affected colony dog on a fine scale. AM-colony dogs are cd-affected dogs from a pedigree segregating the disease originated by cross-breeding a purebred, cd-affected AM to a beagle-mix dog (details about the colony have been described by Sidjanin and colleagues [13]). Primers designed to amplify genomic regions within the neighboring genes successfully amplified fragments from an affected dog within the genes solute carrier family 7 (cationic amino acid transporter, y + system) member 13 (*SLC7A13*), WW domain containing E3 ubiquitin protein ligase 1 (*WWP1*), cyclic nucleotide binding domain containing 1 (*CNBD1*), and matrix metallopeptidase 16 (*MMP16*) (Figure 1; primer pairs 1, 2, 41–43, and 44–46 in Table S1A in Additional file 1). Primers, evenly distributed between *WWP1* and *CNBD1* genes, amplified the proximal (primer pairs 3–9) and distal (primer pairs 20–40) regions and yet failed to amplify the region CFA29: 35,699,379-36,103,729 (primer pairs 10–19) in the cd-affected dog (Table S1A in Additional file 1).

To refine the breakpoints of the deletion, a forward primer from the proximal end of the interval (“left end 7 F” in pair 9, Table S1A in Additional file 1) was paired with two reverse primers from the distal end (“right set 4 1R” in pair 19 and “right set 4 1.5R” in pair 20, Table S1A in Additional file 1). Both of these primer pair combinations are more than 406 kb apart in a normal dog (CFA29: 35,698,350-36,104,507 and CFA29: 35,698,350-36,105,809) and are expected to fail in a PCR reaction. Results confirm this hypothesis: both primer pairs failed to amplify DNA from a normal dog. However, in the cd-affected AM-colony dog the first primer pair (left_end_7F/right_set4_1R) amplified a 1,336 bp fragment, and the second primer pair (left_end_7F/right_set4_1.5R) amplified a fragment larger than 2,600 bp. Sequencing the shorter fragment revealed a 404,820 bp deletion (position 35,699,378-36,104,197). The deletion starts in the first coding exon of *CPNE3* (Copine III) and includes the rest of the gene, the complete *CNGB3* gene, and exons 1–4 of the *CNBD1* gene (Figure 1).

**Figure 2:**
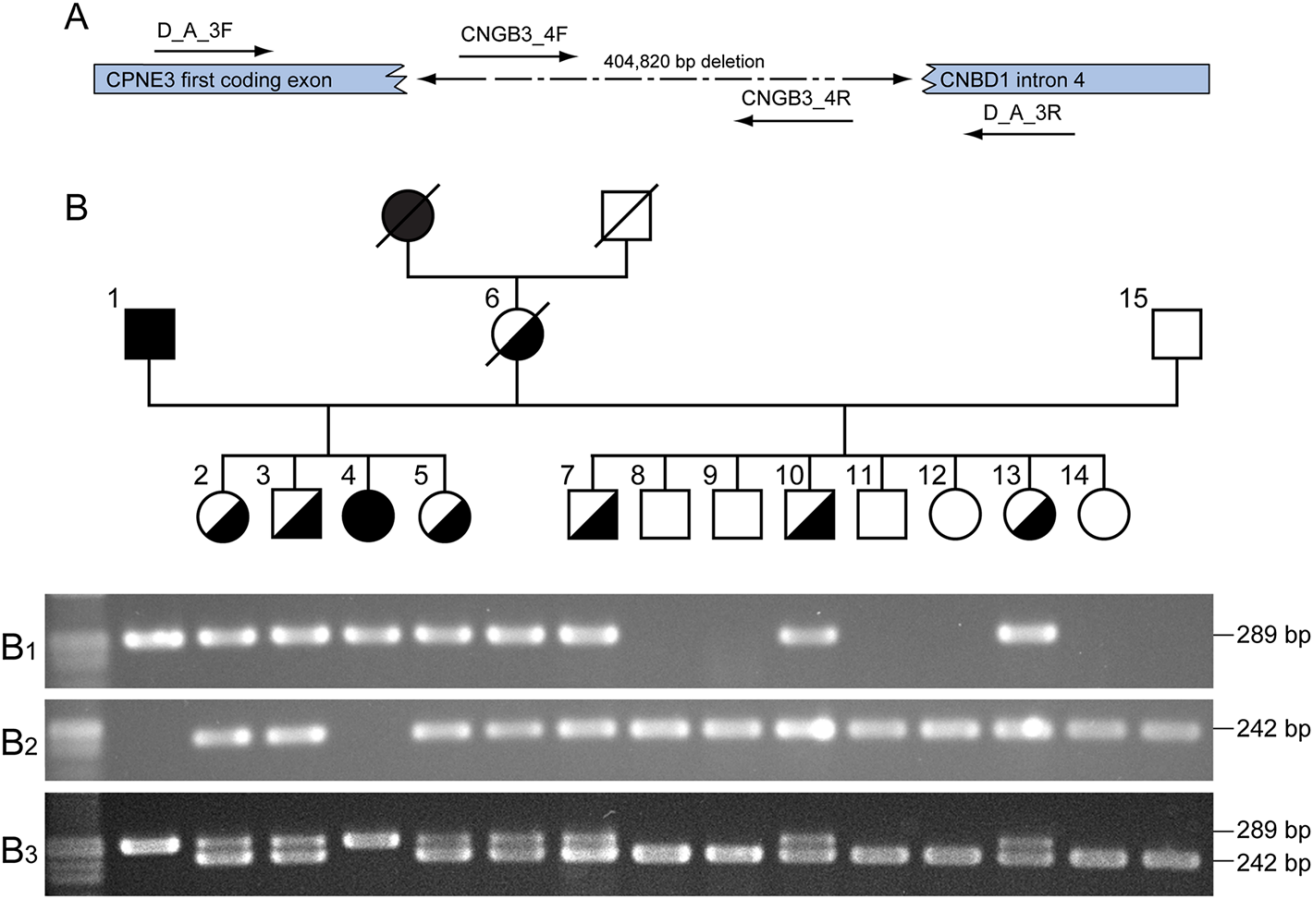
***cd*-PCR based test. A.** A schematic presentation of the test: primers flanking the deletion (D_A_3F/D_A_3R), combined with primers within the deleted sequences (CNGB3_4F/CNGB3_4R) were designed to amplify an affected and a normal chromosome, respectively. Primers flanking the deletion would not amplify a normal chromosome since they are more than 404 kb apart, but would give a product of 289 bp from an affected chromosome. The primer pair within the deletion would only amplify a normal chromosome and result in a 242 bp fragment. Dashed line represents the deleted region. **B.***cd*-PCR based test results are observed in a colony pedigree genotyped for the deletion. A PCR product of 289 bp molecular weight is observed when using primers flanking the deletion (D-A-3 F/3R) in affected dogs (B1, dogs number 1 and 4), and in carrier dogs (B1, dogs number 2, 3, 5, 6, 7, 10, and 13). A PCR product is observed when using primers within the deleted sequence (CNGB3_4F/4R) in normal dogs (B2, dogs number 8, 9, 11, 12, 14, and 15) and in carrier dogs (B2, dogs number 2, 3, 5, 6, 7, 10, and 13). When using both primer pairs in a multiplex reaction, only one band of 289 bp long is observed in affected dogs (B3, dogs number 1 and 4), two bands are observed in carrier dogs (B3, dogs 2, 3, 5, 6, 7, 10, and 13) and only one band of 242 bp long is observed in the normal dogs (B3, dogs 8, 9, 11, 12, 14, and 15).

Once the breakpoints of the deletion were established, a test was designed to genotype normal, carrier and affected dogs by using primers flanking the deletion, which would only amplify the affected allele, combined with primers within the deleted sequence, which would only amplify the normal allele (Figure 2A; see Methods for details). A subset of 15 colony dogs segregating the disease was genotyped (Figure 2B). Two affected dogs (dogs 1 and 4) were positive for the mutated allele PCR (Figure 2B-B1) and negative for the wildtype allele PCR (Figure 2B-B2). One normal dog known to not carry the mutated allele (dog 15) was negative for the mutated allele PCR and positive for the wildtype allele PCR. Three obligated carriers (dogs 2, 3, 5 and 6) were positive to both PCR reactions. Dogs 7–14 were all phenotypically normal, and the genetic test identified dogs 7, 10 and 13 as carriers, and the rest as homozygous wildtype. These primers were also used together in a multiplex PCR (Figure 2-B3). We extended our screening to include samples from ten purebred AMs we had in our laboratory. Among these ten animals were one dog diagnosed with day-blindness at 7 months of age and its parents, one dog diagnosed with day-blindness, one dog suspected of having progressive retinal atrophy (PRA), and five dogs not known to exhibit day-blindness. The parents of the affected dog were genotyped as carriers of the cd-mutation and their offspring as homozygous affected. The rest of the dogs were all genotyped as homozygous normal, including one day-blind AM (data not shown).

### Phenotype and genotype characterization of *CNGB3*-deletion-achromatopsia in a miniature Australian shepherd (MAS)

One affected, male-neutered MAS from a family of day-blind dogs was initially presented at 5 months and then 12 months of age for visual deficits in bright daylight. The authors were made aware of other day-blind dogs related to the MAS in this study but were unable to obtain more information or blood samples from the breeder. Visual deficits were first noted at 3-months of age. This dog collided with objects in bright daylight outside but his vision greatly improved under dim light conditions, such as at night. The dog was unable to locate a ball while playing fetch in daylight unless he heard it bounce nearby or smelled it (Movie in Additional file 2). No other health problems were reported by the owner.

**Figure 3:**
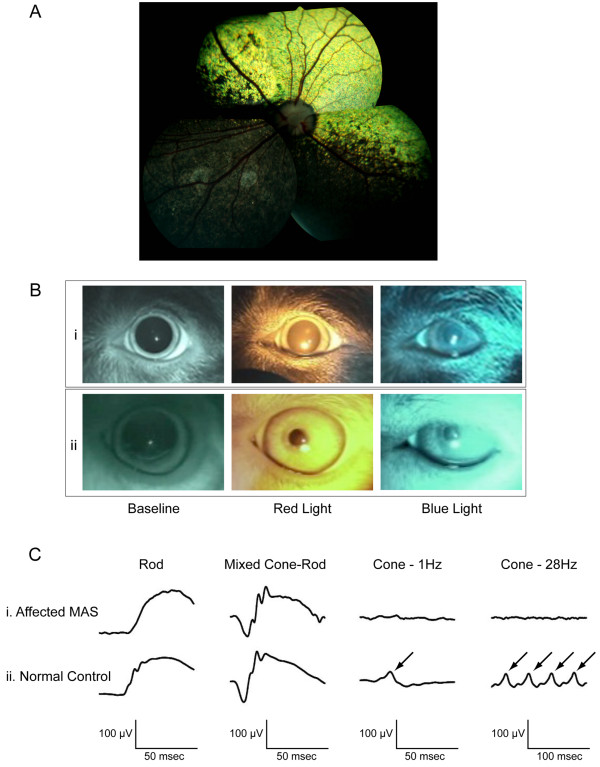
**Ocular fundus, chromatic pupillary light reflexes, and electroretinogram of a day-blind MAS. A.** Compiled image of the normal ocular fundus of the day-blind MAS. **B.** Chromatic pupillary light reflexes. i. Day-blind MAS: Normal pupillary constriction was observed with the blue light stimulus (480 nm, 200 kcd/m^2^), whereas no pupil constriction was observed with the red light stimulus (630 nm, 200 kcd/m^2^) of the same light intensity. ii. Normal canine: Complete pupillary constrictions were elicited with both bright red and blue light. **C.** Electroretinograms. Compared to a normal, age-matched, mixed-bred colony dog (ii) with normal cone-mediated ERG responses (ii, black arrows), the recordings of the day-blind MAS (i) revealed normal rod- but absent cone-mediated retinal function.

A complete ophthalmic examination, including slit lamp biomicroscopy, indirect ophthalmoscopy, applanation tonometry, and streak retinoscopy, was performed and found to be within normal limits (Figure 3A). Systemically, the dog appeared to be healthy as assessed by complete physical examination, complete blood count, and chemistry panel. As a mean for preliminary assessment of retinal dysfunction, chromatic pupillary light reflexes were evaluated qualitatively by use of diffuse red (630 nm – stimulation of long- and medium- wavelength absorbing cones) and blue (480 nm – stimulation of melanopsin-containing intrinsically photosensitive retinal ganglion cells) lights of high intensity (200 kcd/m^2^) [22-24]. The severely reduced pupillary reflexes to the red light with normal pupillary reflexes to the blue light were suggestive of lost cone but normal inner retinal function (Figure 3B). Electroretinography revealed an absence of cone-mediated function, whereas the rods continue to function normally (Figure 3C). Combined with the other clinical findings these functional abnormalities were most suggestive of achromatopsia.

**Figure 4:**
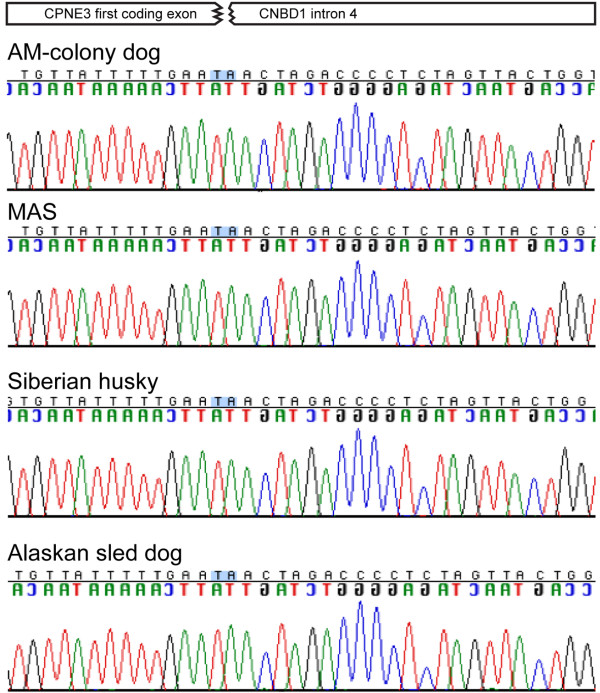
**Alignment of the cd-mutation in four breeds.** Alignment of the sequences obtained from cd-affected alleles of four different breeds: AM, MAS, Siberian husky, and Alaskan sled dog. All four breeds had the same 404,820 bp deletion with identical breakpoints between the first coding exon of *CPNE3* and intron 4 of *CNBD1*.

Genomic DNA was extracted from a whole blood sample to determine if the dog was affected by a mutation in the *CNGB3* gene using the previously described multiplex PCR test. The PCR results confirmed a deletion within the same locus: amplification was positive while using the primers specific to the mutated allele (D_A_3F/D_A_3R in Figure 2A), and negative while using the primers specific to the wildtype allele (CNGB3_4F/CNGB3_4R in Figure 2A). Sequencing of the positive product revealed that the animal was homozygous for the complete genomic deletion of the *CNGB3* gene with the same breakpoints as in the cd-affected AMs (Figure 4).

### Screening for the mutated allele in other canine breeds

The presence of the *CNGB3*-deletion mutation in a MAS, a breed not known to be closely related to the AM [20], suggested that other breeds, more closely related to the AM, might carry the affected allele. We also wanted to expand our screening in the MAS population in an attempt to evaluate the frequency of the affected allele. For that, we collected DNA samples from dogs of five different breeds and unrelated to each other for at least three generations (no parents or grandparents in common): MAS (49 dogs), Australian shepherd (118 dogs), Siberian husky (57 dogs), Samoyed (60 dogs), and Alaskan sled dogs (38 dogs). The screening was done on pooled samples (see Methods for details). One Siberian husky and three Alaskan sled dogs were identified as carrying the cd-affected allele. All three sled dogs were from a subgroup of distance runners and did share a common ancestor four and five generations back. The Siberian husky and the three sled dogs’ mutated alleles were sequenced and aligned to the AM and MAS, showing identical breakpoints of the deletion (Figure 4). No new mutated alleles were found in the MAS group, and none were found in the Samoyed group or the Australian shepherds. Allele frequencies in each breed sample were estimated as 2% in the MAS, 0.877% in the Siberian huskies, and were undetectable in the Samoyeds and Australian shepherds. A larger number of sled dogs will be required to get a better estimation of the mutated allele in that group of dogs.

### Establishing identical by descent (IBD) in the canine breeds affected by *CNGB3*-deletion-achromatopsia and determining the minimal linkage disequilibrium (LD)

To further support the idea that the affected alleles observed in the three different breeds are IBD, we sought to genotype the DNA flanking the deletion, and determine if all affected alleles share the same genetic variations. Seven primer pairs were used to amplify a region up to 5.5 kb upstream and up to 13.7 kb downstream of the mutation (Table S1B in Additional file 1). The genotypes were compared between a purebred, cd-affected AM and three AMs not affected with *CNGB3*-deletion-achromatopsia (one of the three unaffected AMs was an obligated carrier, the parent of the affected dog). We also genotyped a cd-affected AM-colony dog, a purebred affected and unaffected MAS, and a normal boxer. Haplotype analysis identified a shared haplotype between the cd-affected AM and the cd-affected MAS, composed of 34 polymorphisms in a ~20-kb segment (Table S2 in Additional file 1). This haplotype was not observed in the normal MAS or the AMs that did not carry the affected allele.

**Figure 5:**
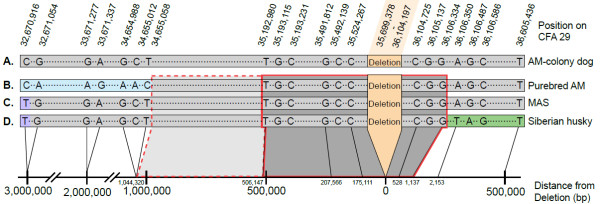
**Schematic presentation of the shared haplotype across five dogs from three different breeds.** Alignment of the affected haplotype observed in five dogs carrying the cd-affected chromosome. Single nucleotide polymorphisms (SNPs) are represented by letters, and their location is denoted in the header. The minimal shared haplotype is boxed in red (0.5 Mb) and the maximum shared haplotype is marked with the dashed extended box (1.04 Mb). **A.** AM-derived colony dog affected with cd. **B.** Affected haplotype observed in a homozygous state in a purebred AM affected with cd and in a heterozygous state in its parent*.***C.** MAS affected with cd. **D.** Siberian husky carrying the cd-affected chromosome. Only the affected chromosome is presented in the diagram. Distances from the mutation are represented in the bottom scale and are drawn to scale.

To further determine how far the deletion-associated haplotype expands, 32 more amplicons surrounding the mutation were added (Table S1C in Additional file 1). One hundred and thirteen additional polymorphisms were identified, to total a 147 polymorphism haplotype in a 3.93 Mb interval (Table S3 in Additional file 1). The fine-mapping was carried out on five dogs: one carrier AM and its affected offspring, one affected AM-colony dog, one affected MAS, and one carrier Siberian husky. A normal boxer was also genotyped as a control reference. These five dogs represented eight affected chromosomes from three different breeds. The genotypes showed that the purebred cd-affected AM and its carrier parent have a different haplotype from the AM-colony dog at the proximal end (32,670,916-34,655,058), suggesting a shared haplotype of 1.0–1.5 Mb (Table S3 in Additional file 1, and Figure 5A and B). The cd deletion allele from the purebred AMs and the cd-affected MAS shared the same 1.0–1.5 Mb haplotype (Figure 5B and C). The affected chromosome carried by the Siberian husky had the same proximal haplotype as the MAS but the distal end was different than both the AM and the MAS starting at position 36,106,350 (Figure 5D). This reduces the shared haplotype across all breeds to 0.5 Mb. The two SNPs defining the minimal LD across all three breeds were 34,655,058 and 36,106,350 (1.04 Mb of sequenced interval on the affected chromosome). Within that interval, a 0.5-Mb segment at the proximal end was not genotyped (between 34,655,058 and 35,192,980; Figure 5, dashed frame). Therefore the shared affected haplotype across all breeds is a minimum of 0.5 Mb and a maximum of 1.04 Mb (Figure 5). The shared haplotype is composed of 53 polymorphisms (Table S3 in Additional file 1, red box). This shared haplotype supports the idea that the mutated alleles in the AM, Siberian husky and the MAS are IBD. Within the 0.5 Mb-interval, one microsatellite was identified, showing different alleles between the affected chromosomes (Table S3 in Additional file 1, polymorphisms number 77). This allelic difference most likely represents a mutation rather a recombination event, due to high mutation rate of microsatellites, and has not been included in the haplotype analysis.

## Discussion

*CNGB3*-achromatopsia is the most common form of achromatopsia in humans [9,12]. In dogs, two different *CNGB3* mutations were identified that result in the achromatopsia phenotype [13]. One is the D262N missense mutation in German shorthaired pointers, while the other is a genomic deletion of *CNGB3* found in the AM [13]. Both of these mutations lead to the same disease phenotype with complete loss of cone function [14]. In our study, we found that affected AMs have a 404,820 bp deletion containing three known genes, *CPNE3, CNGB3,* and *CNBD1*. Since dogs with either the D262N missense mutation or the genomic deletion of *CNGB3* show very similar disease phenotype, the loss of the *CPNE3* and *CNBD1* genes does not appear to have phenotypic consequences. CPNE3 is part of a family of proteins known as the copines. They all share a similar structure and interact with a range of cell signaling and cytoskeletal proteins in response to increases in intracellular calcium [25,26]. They are known to be responsible for calcium-dependent phospholipid binding [25,26]. *CNBD1* has not been fully characterized. Future studies comparing the two types of *CNGB3* mutations may shed light on the function and importance of *CPNE3* and *CNBD1* by identifying subtle phenotypic differences.

One day-blind AM was genotyped normal for the *CNGB3*-deletion mutation. We do not have pedigree information or access to DNA samples from its close relatives; therefore, with these limitations we can only suggest heterogeneity for day-blindness in the AM breed, though non-genetic basis for that particular dog is not excluded. Seddon and colleagues [27] found genetic heterogeneity of day-blindness in AMs in Australia.

We also report the occurrence of achromatopsia in a MAS based on the identical classical clinical phenotype and genomic *CNGB3* deletion that was reported in the AM. Pooled-sample screening identified other arctic breeds closely related to the AM carrying the affected allele: one Siberian husky and three Alaskan sled dogs. We are not aware of any reports about day-blindness in these arctic breeds; however, the occurrence of achromatopsia would not be surprising based on our findings.

The presence of the same large genomic *CNGB3* deletion in several related and unrelated canine breeds suggests that these mutated alleles are IBD. To further confirm this idea, we expanded the locus genotyping to a 3.93 Mb interval. One hundred and forty seven SNPs were identified and confirmed an identical shared affected haplotype across the three breeds, a segment of 0.5–1.04 Mb in size. Comparing haplotypes of large segments of DNA between unrelated dogs gives the opportunity to observe historical recombination, instead of meiosis recombination within a specific pedigree. This is a powerful tool when the dogs that are chosen are the least related to each other within a breed, as well as across different breeds. The shared affected haplotype observed in the purebred affected AM was reduced from 3.53 Mb segment to 1.0–1.5 Mb when compared to affected MAS, and then further reduced to 0.5–1.04 Mb when compared to a third breed, the Siberian husky.

Linkage disequilibrium (LD) mapping has become a useful tool for genomic studies and has been previously used in studies of inherited canine retinal diseases [28-30]. As exemplified in the case study presented here, dogs which share the same ancestral mutated chromosome, also share the same haplotype within a region flanking the mutation, a region that is in LD with the disease. This is especially powerful when the same disease is observed in more than one breed [28,31,32]. The results in this study suggest that *CNGB3* deletion is an ancestral mutation that originated from a dog that served as a common founder. Although most of the dogs carrying the achromatopsia mutation possess an arctic lineage, we have also found it in the MAS whose distinct physical characteristic, functional, and genetic background suggest a different breed origin. The MAS breed was developed in the late 1960s as smaller Australian shepherds were selectively bred in order to achieve the desired size. Contrary to the name of the breed, Australian shepherds were relatively recently developed in the western United States in the 19^th^ and early 20^th^ century. In contrast, the AM is thought to be among the more ancient breeds [20]. During the time of the Klondike Gold Rush in the 19^th^ century, the AM became valuable and was frequently crossbred with other breeds, possibly including the Australian shepherd. Achromatopsia was first observed in an inbred strain of the AM in the 1960s [15-18].

The Alaskan sled dogs are a population of dogs with a northern breed ancestry and were developed through the selection and breeding of several dog breeds based on their athletic abilities. They are mixed breed dogs comprised of several different lineages and can be separated into two clusters, sprint and distance, based on their racing style [33]. It was found that the AM and Siberian husky contributed to enhanced endurance, the pointer and saluki contributed to enhanced speed, and the Anatolian shepherd contributed to work ethic [33]. The presence of both AM and Siberian husky in their ancestry suggests that the mutated cd-allele in these sled dogs could have been contributed by either breed. While it is established that the AM, Siberian husky, and Alaskan sled dog are closely related and belong within the same ancestral cluster [20,21,33], the relation of AM to MAS is less clear. Therefore, based on breed history, it is difficult to determine when a mixture between any of these breeds occurred that introduced the mutation.

There are similar reports of the founder effect in other genetic diseases in dogs. Founder effect may be more obvious in more closely related breeds. Examples include the mutation in *ADAMTS17*, which causes primary lens luxation in many breeds, most of which are terriers or breeds with terrier co-ancestry [32], and a 7.8-kb deletion in the non-homologous end-joining factor 1 (*NHEJI*1) that is responsible for collie eye anomaly in several breeds, most of which fall into a cluster of collie-like dogs [34]. Further example includes multidrug sensitivity, which is caused by a mutation in the canine multidrug resistant gene *MDR1*: Several collie-related breeds and two sight hounds, not expected to share collie ancestry, were found to segregate the affected allele which was determined to be IBD since it was conserved among these affected breeds [35]. Another example is the mutation causing progressive rod-cone degeneration (prcd) in over 20 dog breeds representing all the breed groups defined by the American Kennel Club (herding, hound, working, terrier, toy, sporting and non-sporting as well as miscellaneous) [28]. This apparent disparate association is similar to our findings of the identical *CNGB3* mutation in the MAS and three arctic breeds.

Day-blindness is a rather rare finding in canines with only *CNGB3*-achromatopsia having been thoroughly characterized. Based on eye certification data [36] and our DNA testing results over the past 20 years, we suspect that *CNGB3*-achromatopsia has become rare or even non-existent in North American AMs, but it is still reported in Australia [27,37]. Except for a transgenic *CNGB3*-knockout mouse [38], the dogs discussed here represent the only known animal model of *CNGB3*-achromatopsia, the most common form of the disease in humans [9,12]. Naturally occurring achromatopsia based on mutations in other genes have been described in Awassi sheep (*CNGA3*) [39,40] and in mice (*CNGA3*, *GNAT2* and *PDE6C*) [41-43]. These animals provide valuable models for translational research, including the development of new therapies [14,41,44].

## Conclusion

We found genomic *CNGB3* deletion in breeds not previously known to carry the mutation and showed that there is a founder effect. Our findings suggest that this mutation and the resulting day-blindness are likely present in other canine breeds that have not yet been identified with this disorder, such as the Siberian husky and other arctic breeds. We also reported the occurrence of *CNGB3*-deletion-achromatopsia in a new canine breed, the MAS.

## Methods

### Determining the deletion breakpoints and establishing a diagnostic test

DNA was extracted from blood of a normal and an AM-colony cd-affected dog using QIAmp DNA Blood Mini Kit (Qiagen, Valencia, CA, USA), following manufacturer protocol. This research colony is maintained at the Retinal Disease Studies Facility of the University of Pennsylvania (Kennett Square, PA, USA) and supported by the National Eye Institute, NIH (R01-EY006855) and a Foundation Fighting Blindness (FFB) Center grant (see review on the establishment of the colony by Sidjanin and colleagues [13]). All procedures involving animals were done in compliance with the ARVO Statement for the Use of Animals in Ophthalmic and Vision Research and approved by the University of Pennsylvania IACUC. Primers were designed to amplify genomic fragments within the predicted region where the deletion had taken place, based on the CanFam 2 assembly (http://genome.ucsc.edu/cgi-bin/hgGateway; Table S1 in Additional file 1). Products were evaluated for presence/absence and size by comparing the two samples. Targeted sequences were initially chosen within the *CNGB3* gene neighboring sequences and subsequently narrowed down to pinpoint the breakpoints. PCR products were sequenced using the Applied Biosystems Automated 3730 DNA analyzer (Applied Biosystems, Foster City, CA, USA), and analyzed using Sequencher 4.2.2 software (Gene Codes Corporation, Ann Arbor, MI, USA).

After establishing the breakpoints, primers were designed to identify cd-affected, cd-carrier and normal dogs by PCR, amplifying the normal and mutated alleles in separated reactions as well as in one multiplex PCR reaction. One primer pair (CNGB3_4F: CGACTCTATCTCCTGTGGCTCT, CNGB3_4R: ATTTGTCAGTTTCTGCTTCTCC), which is located within the deleted sequence and therefore is specific to the normal allele, would amplify a 242 bp fragment from a normal chromosome. The second primer pair (D_A_3F: CAAAGTCGGACCCTTTATGTG, D_A_3R: GGCCAAATAGTAGTTCCTGAAA), which is located in sequences flanking the deletion and therefore is specific to the affected allele, would amplify a 289 bp fragment only from an affected chromosome, since on a normal chromosome these primers are more than 404 kb apart (Figure 2). A carrier dog would present both products. PCR products were visualized on a 1.8% agarose gel stained with ethidium bromide. Ten purebred AMs were screened. Those included one dog diagnosed with day-blindness and its parents, one PRA suspicious dog, one dog with day-blindness, and five dogs not known to have day-blindness. The DNA of one day-blind MAS was tested for the presence of the *CNGB3*-deletion mutation. AM-derived cd-affected and cd-carrier colony dogs, as well as normals, were used as controls. PCR products using D_A_3F/D_A_3R primers were sequenced and aligned for comparison, using Sequencher® 4.2.2 Software.

### Clinical examination of dogs

Day-blindness was diagnosed by behavioral vision testing under photopic and scotopic light conditions. In colony dogs, the visual performance under different light conditions was evaluated by use of an obstacle-avoidance test as previously described [45]. For the privately owned MAS behavioral vision testing was performed by observing the dog maneuver around objects and its ability to find toys under different light conditions. The retinal function of this day-blind MAS was first evaluated by assessment of the pupillary light reflexes with diffuse white, red, and blue light in a dark room. The responses to white light were elicited with a finoff transilluminator (Welch Allyn, Skaneateles Falls, NY, USA) and a slit lamp biomicroscope (Kowa SL-14; Kowa Co. Ltd., Tokyo, Japan). The Melan-100 system (Biomed Vision Technologies, Ames, IA, USA) was used as a source for the colored light. This device consists of a bright (200 kcd/m^2^) red (630 nm) and blue (480 nm) LED light for the stimulation of canine long- and medium-wavelength-absorbing (L/M) cones and melanopsin-containing intrinsically photosensitive retinal ganglion cells (ipRGCs), respectively [22]. Constriction of pupil was assessed during illumination with each light for 10 seconds with a 30-second recovery period between tests.

In order to perform ophthalmic examinations, the pupils were dilated by pharmacologic mydriasis using 1% tropicamide ophthalmic solution. The anterior segments of the dogs’ eyes were examined by slit-lamp biomicroscopy (Kowa SL-14). Fundic examinations were performed with a portable binocular indirect ophthalmoscope (Keeler All Pupil II; Keeler Instruments, Broomall, PA, USA) and a condensing lens (Pan Retinal 2.2D; Volk Optical, Mentor, OH, USA). Refraction occurred by streak retinoscopy in both the horizontal and vertical meridians using the hand held Welch Allyn retinoscope and Luneau Skiascopy Rack Set (Wilson Ophthalmic, Mustang, OK). Ocular surfaces were anesthetized with 0.5% proparacaine ophthalmic solution for measurement of the intraocular pressures by use of an applanation tonometer (Tono-Pen XL; Reichert, Depew, NY, USA).

### Electroretinography

Rod and cone photoreceptor mediated retinal function was evaluated by electroretinography. Standard Ganzfeld scotopic and photopic electroretinograms (ERGs) were recorded from the purpose-bred colony dogs under general anesthesia using previously described testing procedures [14]. In the MAS described in this study, the pupils were dilated with 1% tropicamide ophthalmic solution. ERGs were recorded under sedation with intravenous acepromazine maleate (0.01 mg/kg) and butorphanol tartrate (0.1 mg/kg). Two subdermal platinum needle electrodes (Grass Technologies, Astro-Med, Inc., West Warwick, RI, USA) were placed on the forehead between the two eyes (reference) and over the occipital protuberance (ground). The ocular surface was anesthetized with 0.5% proparacaine ophthalmic solution before the placement of a Kooijman/Damhof ERG lens (Acrivet, Henningsdorf, Germany). This lens combines the corneal contact lens electrode for recording with a white LED light source for retinal stimulation. The responses were recorded by use of the RETIport ERG system (Acrivet, Henningsdorf, Germany). Single rod and mixed rod-cone responses were recorded after 20 minutes of dark adaptation by use of single flash stimuli of 0.03 cd.s/m^2^ and 3 cd.s/m^2^, respectively. A total of 20 responses were averaged each at intervals of 10 and 2 seconds, respectively. A 10 minute light adaptation was completed with light from the Kooijman/Damhof ERG lens (25 cd/m^2^). Subsequently, single cone and 28-Hz cone flicker responses were recorded using flash stimuli of 3 cd.s/m^2^ intensity. Twenty traces were averaged for both photopic responses at 2-second intervals.

### Screening other breeds for the presence of the genomic *CNGB3*-deletion

Blood was collected from all the dogs and genomic DNA extracted as described above. In addition to the testing of the privately owned, day-blind MAS, the following strategy was pursued to screen canine breeds for the *CNGB3*-deletion mutation: DNA samples were pooled for PCR reaction in the Australian shepherds (118 dogs from 22 countries, in 12 pooled samples), MAS (49 dogs from six countries, in five pooled samples), Siberian huskies (57 dogs from 13 countries, in six pooled samples), Samoyeds (60 dogs from seven countries, in six pooled samples) and Alaskan sled dogs (38 dogs; 23 sprints runners and 15 distance runners). Two pooled controls were used as a proof of principle of the sensitivity of the pooling strategy: one cd-affected dog pooled with nine normal dogs, and one cd-carrier dog pooled with nine normal dogs. Within each breed, dogs were chosen such that there were no grandparents in common.

### Establishing IBD status with the genomic *CNGB3*-deletion in the cd-affected dogs and determining the minimal linkage disequilibrium (LD)

The affected haplotype surrounding the deletion point was established by sequencing PCR amplicons using seven primer pairs (Table S1B in Additional file 1). Eight dogs were sequenced and compared to confirm IBD: one mixed breed cd-affected AM-colony dog [13], one purebred cd-affected AM, one purebred cd-carrier AM, two purebred AMs not carrying the cd mutation, one normal boxer, one normal MAS, and the day-blind MAS affected by cd.

In order to determine the expansion of the cd-associated haplotype with the genomic *CNGB3*-deletion, a set of 32 primer pairs (Table S1C in Additional file 1) was designed to amplify regions within a 3.93-Mb interval flanking the mutation (3.03 Mb distal to the mutation: 32,670,680 to mutation, and 0.5 Mb proximal to the mutation: mutation to 36,605,630). Five dogs carrying cd-affected alleles from three different breeds were genotyped: one AM cd-carrier, one AM cd-affected, one cd-affected AM-colony dog, one cd-affected MAS, and one cd-carrier Siberian husky. A normal boxer was sequenced to serve as a reference.

## Abbreviations

ADAMTS17: A disintegrin and metalloproteinase with thrombospondin motif 17; AM: Alaskan malamute; cd: Cone degeneration; CFA: Canis familiaris autosome; CNBD1: Cyclic nucleotide binding domain containing 1; CNGA3: *Alpha* subunit of cone cyclic-nucleotide gated channel; CNGB3: *Beta* subunits of cone cyclic-nucleotide gated channel; GNAT2: *Alpha* subunit of cone transducin; IBD: Identical by descent; LD: Linkage disequilibrium; MAS: Miniature Australian shepherd; MDR1: Multidrug resistance gene 1; MMP16: matrix metallopeptidase 16; NHEJI1: Non-homologous end-joining factor 1; PDE6C: *Alpha* subunit of cone cyclic guanosine monophosphate (cGMP) phosphodiesterase; PDE6H: *Gamma* subunit of cone cyclic guanosine monophosphate (cGMP) phosphodiesterase; PRA: Progressive retinal atrophy; prcd: progressive rod-cone degeneration; SLC7A13: solute carrier family 7 (cationic amino acid transporter, y + system) member 13; WWP1: WW domain containing E3 ubiquitin protein ligase 1

## Competing interests

GMA and SPK have a financial interest in the development of genetic testing/diagnostic test and own equity in a company (OptiGen, LLC, Ithaca, NY, USA). The remaining authors have declared that no conflict of interest exists.

## Authors’ contributions

CYY and AMK carried out all clinical examination and diagnostics, and wrote the initial draft of the manuscript. OG carried out the molecular genetics study and drafted part of the manuscript. AVK participated in deletion characterization and edited the manuscript. DH, AK, and HJH collaborated in phenotyping dogs and sample collection. SPK participated in the design and coordination of the study and helped draft the manuscript. CYY, OG, AMK, GMA conceived of the study and participated in its design and coordination. All authors read and approved the final manuscript.

## Supplementary Material

Additional file 1: Table S1 Primer names, sequences and locations. A. Primers used to establish the breaking points of the cd mutation and the corresponding presence or absence of the PCR product in cd-affected dog. In bold are the primers flanking the deletion used to identified the sequence of the affected chromosome and identify the accurate breaking point in the DNA. B. Primers used to establish the affected haplotype linked to the mutation in a ~20 Kb interval. C. Primers used to establish the minimum LD between the affected alleles in the AM, the MAS and the Siberian husky in a 3.93 Mb interval. **Table S2** Affected and normal haplotypes in the *cd*-locus in AM and MAS. Abbreviations: del, deletion; wt, wildtype; mut, *CNGB3*-deletion mutation. **Table S3** Genotype results of five dogs to total eight cd-affected chromosomes from three different breeds. Boxed in red is the minimal LD, 0.913-1.45 Mb long and observed across all affected chromosomes. Highlighted in colors are the informative SNPs with different colors to each allele. Abbreviations: del, deletion; wt, wildtype; mut, *CNGB3*-deletion mutation. Click here for file

Additional file 2 **Movie showing the cd-affected MAS trying to fetch a ball under day-light conditions.** The dog could only find the ball by smell, not by sight. Click here for file
