# Supplementary material for: Genomic deletion of CNGB3 is identical by descent in multiple canine breeds and causes achromatopsia
Source: BMC Genet. 2013 Apr 20;14:27. doi: 10.1186/1471-2156-14-27 (PMC3639114; doi:10.1186/1471-2156-14-27)
Supplement: Additional file 1: Table S1 — Primer names, sequences and locations. A. Primers used to establish the breaking points of the cd mutation and the corresponding presence or absence of the PCR product in cd-affected dog. In bold are the primers flanking the deletion used to identified the sequence of the affected chromosome and identify the accurate breaking point in the DNA. B. Primers used to establish the affected haplotype linked to the mutation in a ~20 Kb interval. C. Primers used to establish the minimum LD between the affected alleles in the AM, the MAS and the Siberian husky in a 3.93 Mb interval. Table S2 Affected and normal haplotypes in the cd-locus in AM and MAS. Abbreviations: del, deletion; wt, wildtype; mut, CNGB3-deletion mutation. Table S3 Genotype results of five dogs to total eight cd-affected chromosomes from three different breeds. Boxed in red is the minimal LD, 0.913-1.45 Mb long and observed across all affected chromosomes. Highlighted in colors are the informative SNPs with different colors to each allele. Abbreviations: del, deletion; wt, wildtype; mut, CNGB3-deletion mutation. [file 1471-2156-14-27-S1.docx]

**Table S1: Primer names, sequences and locations. A**. Primers used to establish the breaking points of the cd mutation and the corresponding presence or absence of the PCR product in cd-affected dog. In bold are the primers flanking the deletion used to identified the sequence of the affected chromosome and identify the accurate breaking point in the DNA. **B**. Primers used to establish the affected haplotype linked to the mutation in a ~20 Kb interval. **C.** Primers used to establish the minimum LD between the affected alleles in the AM, the MAS and the Siberian husky dogs in a 3.93 Mb interval.

| **A.** | | | | | | | | | | | | | | | | | | | |
| --- | --- | --- | --- | --- | --- | --- | --- | --- | --- | --- | --- | --- | --- | --- | --- | --- | --- | --- | --- |
| **Primer pair** | **Forward primer name** | | | **Location on CFA29** | | **Forward primer sequence** | | **Reverse primer name** | | **Location on CFA29** | | **Reverse primer sequence** | | | | **Product size** | | **Positive product in cd-affected** | |
| **1** | L3.XAT2 | | | 35419873 35419892 | | TCTCGGTTATGGCGCTTACT | | R4.XAT2 | | 35419673 35419692 | | GGGATGGTACCTGGGAAGTT | | | | 219 | | + | |
| **2** | WWR2 | | | 35541809 35541829 | | CTCCCCCTTTTGAAAATTCTG | | WWL2 | | 35541478 35541498 | | TTCAAATGGTTCTCAGGCATT | | | | 351 | | + | |
| **3** | left_end_1F | | | 35687058 35687078 | | TCAGCACTCCCCAGACTAAAA | | left_end_1R | | 35687543 35687564 | | CAGGATAACAAATCACGGACAA | | | | 507 | | **+** | |
| **4** | left_end_2F | | | 35688558 35688577 | | CAATGTCTGGTTCCTGTGAA | | left_end_2R | | 35688756 35688775 | | CTTGCTTTGATTTTCGCTTC | | | | 218 | | **+** | |
| **5** | left_end_3F | | | 35691972 35691992 | | GCCCTTTTCTCTGTTTGTGTG | | left_end_3R | | 35692291 35692311 | | CAGCAGTCAGTTCCTCTTCAA | | | | 340 | | **+** | |
| **6** | left_end_4F | | | 35695664 35695684 | | GAGAGGATGGCAGTGAGAGTG | | left_end_4R | | 35696207 35696227 | | AAGTGGGCAGAAGAATGTGTG | | | | 564 | | **+** | |
| **7** | left_end_5F | | | 35697195 35697215 | | GTGCTCCGTCTTCACCTAATG | | left_end_5R | | 35697605 35697626 | | GTGTGGCAAAAATCAATACCAG | | | | 432 | | **+** | |
| **8** | left_end_6F | | | 35697809 35697829 | | TTTGTTCCTGTTGTCCCAGAA | | left_end_6R | | 35698330 35698350 | | CCTCACTCCAATCTCCAATCA | | | | 542 | | **+** | |
| **9** | **left_end_7F** | | | 35698350 35698370 | | GGGGGATGTGTGAAAGTTAGG | | left_end_7R | | 35699046 35699066 | | GATTCTCTTGCTGGGATGAAA | | | | 717 | | **+** | |
| **10** | CPNE3exon3F | | | 35699379 35699399 | | CGAGTGGTCAACAGTGGTATG | | CPNE3int4R | | 35699710 35699729 | | GGAAAATGTGGGATTCAAGC | | | | 351 | | **-** | |
| **11** | right_end_1F | | | 35911108 35911128 | | TTCTTGGGTTTTAGCGTGATG | | right_end_1R | | 35911522 35911542 | | AACAACTGCTCTGCTGGACTC | | | | 435 | | **-** | |
| **12** | right_end_2F | | | 35949910 35949930 | | GAGGAGGGAGAGGGATTGATA | | right_end_2R | | 35950460 35950480 | | ACAAGGCGAAACACACAAAGT | | | | 571 | | **-** | |
| **13** | right_end_3F | | | 35980143 35980163 | | CCTGTCACCACATCACCATAA | | right_end_3R | | 35980488 35980508 | | TGCTACATCTCTTCCCCTTGA | | | | 366 | | **-** | |
| **14** | right_end_4F | | | 36017469 36017489 | | CAAGTGGAAGGAACAGGAGGT | | right_end_4R | | 36017934 36017954 | | TTATTTGGATGGAGGGATAGC | | | | 486 | | **-** | |
| **15** | right_end_5F | | | 36025624 36025645 | | AGCAGTCTCCAAACAACAACAA | | right_end_5R | | 36026135 36026155 | | ATTTCCCTCTTTACCCAGCAA | | | | 532 | | **-** | |
| **16** | CNBD1ex2F | | | 36057165 36057185 | | CCAGATGAAAGGGGCAGAATA | | CNBD1ex2R | | 36057564 36057585 | | CCATTAGAGATGAGGCAAGGTC | | | | 421 | | **-** | |
| **17** | CNBD1ex4F | | | 36098323 36098343 | | AGGCGTTGAAAAGGCATACAT | | CNBD1ex4R | | 36098968 36098988 | | TGATGGGGAAGACAGTTGAAA | | | | 666 | | **-** | |
| **18** | right_set3_1F | | | 36103170 36103190 | | CTGTGTTAGGGAGTTCGGTTT | | right_set3_1R | | 36103581 36103600 | | ATGCCTCATTCCATCAAAGG | | | | 431 | | **-** | |
| **19** | right_set4_1F | | | 36103709 36103729 | | GGCTGCTTAAAATCCAAATGC | | **right_set4_1R** | | 36104488 36104507 | | TTCCAAACAACAGAACAGCA | | | | 799 | | **-** | |
| **20** | right_set4_1.5F | | | 36105273 36105293 | | GGGGGTTTCATTACAGAGTCA | | **right_set4_1.5R** | | 36105787 36105809 | | CCCAAGACAAGAAGTCACAGTTT | | | | 537 | | **+** | |
| **21** | right_set4_2F | | | 36105966 36105986 | | TGGCCTCACAAAGGACATTAC | | right_set4_2R | | 36106481 36106504 | | TGCAAACACATCACAGACTACTCA | | | | 539 | | **+** | |
| **22** | right_set4_3F | | | 36106346 36106366 | | ATTTGTCCCTCCTGTTTCACC | | right_set4_3R | | 36106803 36106825 | | TCCTACTCCTCCATCATCTTCAA | | | | 480 | | **+** | |
| **23** | right_set4_4F | | | 36113712 36113732 | | CCTGGTGAAGGGGAAAACATA | | right_set4_4R | | 36114440 36114461 | | GAGGTAAGAGCATTGGCATAGG | | | | 750 | | **+** | |
| **24** | right_set4_5F | | | 36114413 36114435 | | TCTCTGACTGGACTGTTGTTTCA | | right_set4_5R | | 36115117 36115137 | | TTTCCTCAAATCTCCCACCAT | | | | 725 | | **+** | |
| **25** | right_set4_6F | | | 36115217 36115237 | | CAGAAGCCACCTAGAGCACAG | | right_set4_6R | | 36115922 36115942 | | TCCACCCACACTGACACATAA | | | | 726 | | **+** | |
| **26** | right_set4_7F | | | 36115931 36115950 | | AGTGTGGGTGGATGAGCTTC | | right_set4_7R | | 36116435 36116457 | | CTTGGGATGTTCACTGTTACTCA | | | | 527 | | **+** | |
| **27** | right_set3_2F | | | 36116597 36116617 | | TCCTGTGTAGTTTCCGTGGTC | | right_set3_2R | | 36116895 36116915 | | TGAGAGGCAGATAGTCCAAGC | | | | 319 | | **+** | |
| **28** | right_set3_3F | | | 36130304 36130324 | | CACATCTTTGCCCTACTTACG | | right_set3_3R | | 36130610 36130630 | | ATCTCTTGCCCTGACATCTGA | | | | 327 | | **+** | |
| **29** | right_set3_4F | | | 36138013 36138033 | | AAGGAACATCTGGGAACTTTG | | right_set3_4R | | 36138564 36138584 | | GTCATCGGAATGCTAAAACCT | | | | 572 | | **+** | |
| **30** | right_set3_5F | | | 36149912 36149932 | | GTGTTATGTGTGCTCCCCAGT | | right_set3_5R | | 36150429 36150449 | | TGGTTGTCCTTTCAGACCTTT | | | | 538 | | **+** | |
| **31** | right_set3_6F | | | 36157921 36157941 | | GGCTGCTTCTGCTGTTATTGT | | right_set3_6R | | 36158302 36158322 | | TCACTCCCCTACCAAAATGAA | | | | 402 | | **+** | |
| **32** | right_set3_7F | | | 36173297 36173318 | | GGACCTGAGTCTAATCCTTGCT | | right_set3_7R | | 36173763 36173782 | | CTGTCTTCCCACCCCTTACA | | | | 486 | | **+** | |
| **33** | right_set3_8F | | | 36180955 36180975 | | TTGCTTCAAACTTCCCTCCTT | | right_set3_8R | | 36181366 36181389 | | CAAACCTATTCTCACACCACTTTC | | | | 435 | | **+** | |
| **34** | right_set3_9F | | | 36193118 36193138 | | CACATCCATTTCAGGCATACA | | right_set3_9R | | 36193569 36193589 | | TCAATCTTCACACGGTTCTCA | | | | 472 | | **+** | |
| **35** | right_set3_10F | | | 36207022 36207042 | | GTTTCCCTGTGGCTTATTTCA | | right_set3_10R | | 36207531 36207551 | | ATTGACCTCGTCCTTCGTTTT | | | | 530 | | **+** | |
| **36** | right_set3_11F | | | 36216695 36216715 | | TGGAGGACTGAGACAAGAGTG | | right_set3_11R | | 36217101 36217121 | | GGAGGCAGAATAAGACGCAAT | | | | 427 | | **+** | |
| **37** | right_set3_12F | | | 36232689 36232709 | | GAAAAGGGGCAAGGATTTATG | | right_set3_12R | | 36233165 36233185 | | GGAACCTGAATTGTCTGGGTA | | | | 497 | | **+** | |
| **38** | right_set3_13F | | | 36241263 36241283 | | CTAAGGAAATTGGGGTGAAGC | | right_set3_13R | | 36241665 36241685 | | CGCATAGGAAAACAGGTGGTA | | | | 423 | | **+** | |
| **39** | right_set3_14F | | | 36253533 36253553 | | CACTCAGGTCTTACGGGATGA | | right_set3_14R | | 36253936 36253956 | | CTGGGTTTTCCTTGCTATTCC | | | | 424 | | **+** | |
| **40** | right_set3_15F | | | 36260075 36260095 | | CAATGCCAGAAGGACACAGTT | | right_set3_15R | | 36260498 36260518 | | GGAGAGAGTTTGATTGCCTCA | | | | 444 | | **+** | |
| **41** | CNBD1ex5F | | | 36262937 36262959 | | CATCTTAGTGCTGGAAGGAGAAA | | CNBD1ex5R | | 36263261 36263281 | | TCTGTTACCTGTGCTGCCTTT | | | | 345 | | **+** | |
| **42** | CNBD1ex7F | | | 36334703 36334722 | | ACCTGCCATCATCCTTTTTG | | CNBD1ex7R | | 36335114 36335135 | | CCACTTCAACTACCCATCATCA | | | | 433 | | **+** | |
| **43** | CNBD1ex10F | | | 36380247 36380267 | | CTGGAAAAGAGGTTGAGATGG | | CNBD1ex10R | | 36380530 36380550 | | TGGTAACTTGGGAATGAGCAG | | | | 304 | | **+** | |
| **44** | MMP16ex10F | | | 36921723 36921743 | | GTGTGTGTGTTTCCCCCTCTA | | MMP16ex10R | | 36921432 36921452 | | GGGTGTTCCTTTCCTCTTGAA | | | | 312 | | **+** | |
| **45** | MMP16ex10F2 | | | 36921984 36922004 | | AGATGTGTTCAGTGGGCTCTC | | MMP16ex10R2 | | 36921534 36921554 | | GGCTGTGTTGTCCAGTTTGAT | | | | 471 | | **+** | |
| **46** | L2.MMP16/1 | | | 37119010 37119031 | | GCTGCCGTCATCCAAAATTACT | | R2.MMP16/1 | | 37118851 37118870 | | ATTCAGTTTGGCCCGATGTA | | | | 180 | | **+** | |
| **B.** | | | | | | | | | | | | | | | | | | | |
| **Primer pair** | **Forward primer name** | | **Location on CFA29** | | **Forward primer sequence** | | | | **Reverse primer name** | | **Location on CFA29** | | **Reverse primer sequence** | | | | **Product size** | | **SNPs within the amplicon** |
| **1** | rs23525227-8-9F | | 35693780- 35693804 | | GGTATTTTGCTTTTGCTTTGCTTTC | | | | rs23525227-8-9R | | 35694353 35694374 | | ATTGGGAGGGAGAAGTCAGTGC | | | | 595 | | rs23525229  rs23525228  rs23525227 |
| **2** | left_end_7F | | 35698350 35698370 | | GGGGGATGTGTGAAAGTTAGG | | | | left_end_7R | | 35699046 35699066 | | GATTCTCTTGCTGGGATGAAA | | | | 717 | | Novel SNP  rs23530596 |
| **3** | rs23486066F | | 35698518 35698541 | | AAATCTCTTTGTGTGGGACTCTGG | | | | rs23486066R | | 35699042 35699064 | | TTCTCTTGCTGGGATGAAACTCA | | | | 547 | | rs23486066 |
| **4** | rs23486058F | | 35698773 35698794 | | CCACCAACTAAGCCAGCCAGGT | | | | rs23486058R | | 35699330 35699352 | | TCCGACTTTGACCCTACATCCAT | | | | 580 | | rs23486061  rs23486058 |
| **5** | Right16_novel_F | | 36104035 36104061 | | TGTGTTTAGGAGATGAGTTAGCAAGAA | | | | Right16_novel_R | | 36104684 36104708 | | AAGAGGGACTTCACCAGAAAAAGAA | | | | 674 | | Novel SNP |
| **6** | rs23494484F | | 36104649 36104671 | | ATTCTGGAGCCTGGGAAGTCTAA | | | | rs23494484R | | 36105197 36105217 | | ATTTGGGGCACACCCTGAGAG | | | | 569 | | rs23494484  rs23461219 |
| **7** | rs23494487F | | 36106153 36106175 | | TTTTGGGGAGACTTAGGAAATCA | | | | rs23494487R | | 36106633 36106656 | | TGTAGAAGAAAGAGCATTGAACCA | | | | 504 | | rs23494487  rs23492798  rs23494491 |
| **C.** | | | | | | | | | | | | | | | | | | | |
| **Primer pair** | **Forward primer name** | **Forward primer sequence** | | | | | **Reverse primer name** | | **Reverse primer sequence** | | | | | **Product size** | **Amplicon location** | | | | |
| **1** | cdLD25F | TGGCAGCATATGATAAGAGGCAGA | | | | | cdLD25R | | GGGAATTTTAAGTAACATTTCAGTGTG | | | | | 561 | 32,670,680-32,671,240 | | | | |
| **2** | cdLD28F | acccaagcttcactgcatttgac | | | | | cdLD28R | | tgacagaatttgggaaaaggtcag | | | | | 298 | 33,671,148-33,671,445 | | | | |
| **3** | cdLD29F | tgcaaacacacagtcacacaaaca | | | | | cdLD29R | | tgaataacagcaaaagggctcaga | | | | | 487 | 33,671,763-33,672,249 | | | | |
| **4** | cdLD30F | ccttttgctgttattcaacccaca | | | | | cdLD30R | | agttattttgccctgcatagctca | | | | | 767 | 33,672,233-33,672,999 | | | | |
| **5** | cdLD31F | atgtgtgacctagggcgcattact | | | | | cdLD31R | | aactgtgagattgactgtttccttcc | | | | | 583 | 34,173,820-34,174,402 | | | | |
| **6** | cdLD32F | cacatctgagtttctgctgcttcc | | | | | cdLD32R | | tttcccttaggttcctctgcacaa | | | | | 552 | 34,162,299-34,162,850 | | | | |
| **7** | cdLD33F | tgtgcaggaaattgctttgctaga | | | | | cdLD33R | | ggggatatcgaatgtcctcttcct | | | | | 623 | 34,163,011-34,163,633 | | | | |
| **8** | cdLD34F | CATGCAACTCCTGAGGGAAATAGG | | | | | cdLD34R | | GAAGTAAGGCTTCTGCAATCTGTAAGG | | | | | 617 | 34,654,096-34,654,712 | | | | |
| **9** | cdLD35F | ATTGTGCAGTTAGGGACTGGGAAA | | | | | cdLD35R | | TGCCAGGTAGGATGTATTCACCAAT | | | | | 694 | 34,654,610-34,655,303 | | | | |
| **10** | cdLDF1 | ggccagcaaagaatggagagttac | | | | | cdLDR1 | | atggggaaaatggaaaggaagaga | | | | | 556 | 35,192,702-35,193,257 | | | | |
| **11** | cdLDF2 | tccaaaagagagagcaagggaagt | | | | | cdLDR2 | | atggttccaacaagcaggcaagta | | | | | 583 | 35,195,839-35,196,421 | | | | |
| **12** | cdLDF3 | gtagactcccacccattgactcc | | | | | cdLDR3 | | agaagattgcctttgccacgatta | | | | | 432 | 35,196,188-35,196,619 | | | | |
| **13** | cdLDF4 | agtggggtcattaacgcaatcatc | | | | | cdLDR4 | | tgccaacaccctcctcaaagat | | | | | 683 | 35,491,711-35,492,393 | | | | |
| **14** | cdLDF5 | tccttctctggttgaaaatctgaaa | | | | | cdLDR5 | | acttgctcacactgacctgcctgt | | | | | 661 | 35,524,142-35,524,802 | | | | |
| **15** | cdLDF6 | cctttccttagatccttcatccattg | | | | | cdLDR6 | | tgacacagcaacatagtccaaaaataa | | | | | 653 | 35,528,055-35,528,707 | | | | |
| **16** | cdLDF7 | ggcattttctcttttctactttggtga | | | | | cdLDR7 | | actcctctttccctttcttcttgc | | | | | 700 | 35,588,912-35,589,611 | | | | |
| **17** | cdLDF8 | ccaccaaaagtatgcgagagttcc | | | | | cdLDR8 | | gcatcagcaaccAATAAGggcttt | | | | | 553 | 35,610,557-35,611,109 | | | | |
| **18** | cdLDF9 | tggtgccctagcagttccactatt | | | | | cdLDR9 | | tgggatgaaagctaattttggcata | | | | | 593 | 35,612,924-35,613,516 | | | | |
| **19** | cdLDF10 | tcagtggaaggcaataacagagca | | | | | cdLDR10 | | TTCACAGGGCAGAGCAAGgtaac | | | | | 903 | 35,631,917-35,632,819 | | | | |
| **20** | cdLDF11 | caaaggacagcacactACCTCCTC | | | | | cdLDR11 | | gacccttgtcagagatccagcact | | | | | 643 | 35,634,442-35,635,084 | | | | |
| **21** | cdLDF12 | CTCCCAGATTTCCCATCACCTatg | | | | | cdLDR12 | | tccatatacttctcctttgccactg | | | | | 609 | 35,661,346-35,661,954 | | | | |
| **22** | cdLDF13 | tgattgctgtgtttttcatttttgtg | | | | | cdLDR13 | | cagtttggtgctgggttggtg | | | | | 693 | 36,153,371-36,154,063 | | | | |
| **23** | cdLDF14 | taagaatgggcaaaacagagcata | | | | | cdLDR14 | | aactgaaaatgtgagacaagcaagaa | | | | | 700 | 36,174,580-36,175,279 | | | | |
| **24** | cdLDF16 | ttaacgcagaaagagcatgattga | | | | | cdLDR16 | | cagagacacaggcagagggagaag | | | | | 458 | 36,179,606-36,180,063 | | | | |
| **25** | cdLDF17 | ggacatgatttatgagaaccgtgtg | | | | | cdLDR17 | | gccaaacatctggaatatcttttgtc | | | | | 406 | 36,193,557-36,193,962 | | | | |
| **26** | cdLDF18 | tgcgtcttattctgcctcctttatg | | | | | cdLDR18 | | aaatcaatccctaccctcctccat | | | | | 655 | 36,217,103-36,217,757 | | | | |
| **27** | cdLDF19 | gccctgaaagaggcaagaaacag | | | | | cdLDR19 | | gtgaaagagattccccactgagca | | | | | 445 | 36,218,231-36,218,675 | | | | |
| **28** | cdLDF20 | tccttgccattgtctcctgtaaga | | | | | cdLDR20 | | tgtccagaaaagcaaaaacaaaaa | | | | | 493 | 36,293,832-36,294,324 | | | | |
| **29** | cdLDF21 | aattgccgaatgaactttgtaggc | | | | | cdLDR21 | | ggcagaaccattgccttttcttat | | | | | 873 | 36,302,803-36,303,675 | | | | |
| **30** | cdLDF22 | aagttctgagcccagcagaatcat | | | | | cdLDR22 | | atgatggagggccatttagatgtt | | | | | 737 | 36,304,278-36,305,014 | | | | |
| **31** | cdLDF23 | gctaaggtttattgaaggcttgttcc | | | | | cdLDR23 | | ccatattttcatgtagctggcacctt | | | | | 949 | 36,603,989-36,604,937 | | | | |
| **32** | cdLDF24 | aaagaggcctgagaagaaggcaac | | | | | cdLDR24 | | caatcaatgtcctggaatgtgaaga | | | | | 833 | 36,604,798-36,605,630 | | | | |

**Table S2: Affected and normal haplotypes in the *cd*-locus in AM and MAS.** Abbreviations: del, deletion; wt, wildtype; mut, *CNGB3*-deletion mutation.

|  |  |  |  | | Purebred AM | | | | | | | |  | | Purebred MAS | | | |
| --- | --- | --- | --- | --- | --- | --- | --- | --- | --- | --- | --- | --- | --- | --- | --- | --- | --- | --- |
| Location on CFA29 | Distance from deletion in bp | Alleles | Normal Boxer | | *cd*- Normal | | *cd*-normal | | *cd*- carrier | | *cd*- affected | | *cd*- affected AM- colony dog | | *cd*- affected | | *cd*- Normal | |
| 35,693,838 | 5,540 | A/T | A | A | T | T |  |  | T | **T** | **T** | **T** | **T** | **T** | **T** | **T** | T | T |
| 35,693,894 | 5,484 | A/G | A | A | A | G | C | G | C | **C** | **C** | **C** | **C** | **C** | **C** | **C** | G | G |
| 35,693,909 | 5,469 | A/G | G | G | G | G | A | G | A | **A** | **A** | **A** | **A** | **A** | **A** | **A** | G | G |
| 35,693,911 | 5,467 | A/G | A | A | A | G | G | G | G | **G** | **G** | **G** | **G** | **G** | **G** | **G** | G | G |
| 35,693,929 | 5,449 | G/**T** | T | T | G | T | del | wt | del | **del** | **del** | **del** | **del** | **del** | **del** | **del** | G | G |
| 35,694,016 | 5,362 | C/T | C | C | T | C | C | T | C | **C** | **C** | **C** | **C** | **C** | **C** | **C** | T | T |
| 35,694,052 | 5,326 | G/T | G | G | G | G | T | G | T | **T** | **T** | **T** | **T** | **T** | **T** | **T** | G | G |
| 35,694,196 | 5,182 | G/C | C | C | C | C | G | C | G | **G** | **G** | **G** | **G** | **G** | **G** | **G** | C | C |
| 35,694,260 | 5,118 | A/G | G | G | G | G | A | G | A | **A** | **A** | **A** | **A** | **A** | **A** | **A** | G | G |
| 35,698,465 | 913 | G/A | A | A | A | G | G | G | G | **G** | **G** | **G** | **G** | **G** | **G** | **G** | G | G |
| 35,698,634 | 744 | G/C | G | G | G | C | C | C | C | **C** | **C** | **C** | **C** | **C** | **C** | **C** | C | C |
| 35,698,828 | 550 | C/T | C | C | C | C | C | T | C | **C** | **C** | **C** | **C** | **C** | **C** | **C** | C | T |
| 35,698,982 | 396 | A/C | C | C | C | A | A | A | A | **A** | **A** | **A** | **A** | **A** | **A** | **A** | A | A |
| 35,699,124 | 254 | C/T | C | C | C | C | T | T | T | **T** | **T** | **T** | **T** | **T** | **T** | **T** | T | C |
| **35,699,378**  **36,104,197** | 0 |  | wt | wt | wt | wt | wt | wt | wt | **mut** | **mut** | **mut** | **mut** | **mut** | **mut** | **mut** | wt | wt |
| 36,104,213 | 16 | G/A | A | A | A | G | G | G | G | **G** | **G** | **G** | **G** | **G** | **G** | **G** | A | A |
| 36,104,546 | 349 | G/T | G | G | G | T | T | T | T |  |  |  |  |  |  |  | G | G |
| 36,104,725 | 528 | C/T | C | T | C | C | C | C | C | **C** | **C** | **C** | **C** | **C** | **C** | **C** | C | C |
| 36,105,137 | 940 | A/G | A | G | G | G | G | G | G | **G** | **G** | **G** | **G** | **G** | **G** | **G** | G | G |
| 36105138-41 | 941-944 | TCAG del | wt | wt | wt | del | del | del | del | **del** | **del** | **del** | **del** | **del** | **del** | **del** | wt | wt |
| 36,106,334 | 2,137 | G/A | A | G | G | G | G | G | G | **G** | **G** | **G** | **G** | **G** | **G** | **G** | G | G |
| 36,106,350 | 2,153 | A/G | G | G | G | G | A | A | A | **A** | **A** | **A** | **A** | **A** | **A** | **A** | G | G |
| 36,106,365 | 2,168 | T/C | C | T | T | T | T | T | T | **T** | **T** | **T** | **T** | **T** | **T** | **T** | T | T |
| 36,106,487 | 2,290 | A/G | A | G | A | A | G | G | G | **G** | **G** | **G** | **G** | **G** | **G** | **G** | A | A |
| 36,106,526 | 2,329 | A/T | T | T | T | T | T | T | T | **T** | **T** | **T** | **T** | **T** | **T** | **T** | T | T |
| 36,106,586 | 2,389 | C/G | C | G | G | G | C | C | C | **C** | **C** | **C** | **C** | **C** | **C** | **C** | G | G |
| 36,106,948 | 2,751 | A/G | G | G | A | G | A | A | A | **A** | **A** | **A** | **A** | **A** | **A** | **A** | G | G |
| 36,107,071 | 2,874 | C/T | C | T | C | T | T | T | T | **T** | **T** | **T** | **T** | **T** | **T** | **T** | C | C |
| 36,107,199 | 3,002 | C/T | T | T | T | C | C | C | C | **C** | **C** | **C** | **C** | **C** | **C** | **C** | T | T |
| 36,111,465 | 7,268 | A/G | G | G |  |  |  |  | A | **A** | **A** | **A** |  |  |  |  | G | G |
| 36,111,575 | 7,378 | C/T | T | T |  |  |  |  | C | **C** | **C** | **C** |  |  |  |  | T | T |
| 36,111,685 | 7,488 | A/G | G | A |  |  |  |  | A | **A** | **A** | **A** |  |  |  |  | G | G |
| 36,111,794 | 7,597 | A/G | G | G |  |  |  |  | A | **A** | **A** | **A** |  |  |  |  | G | G |
| 36,117,443 | 13,246 | A/G | A | G |  |  |  |  | G | **G** | **G** | **G** | **G** | **G** | **G** | **G** | A | A |
| 36,117,922 | 13,725 | A/G |  |  |  |  | G | G | G | **G** |  |  |  |  | **G** | **G** | A | A |

**Table S3: Genotype results of five dogs to total eight cd-affected chromosomes from three different breeds.** Boxed in red is the minimal LD, 0.913-1.45 Mb long and observed across all affected chromosomes. Highlighted in colors are the informative SNPs with different colors to each allele. Abbreviations: del, deletion; wt, wildtype; mut, *CNGB3*-deletion mutation.

|  |  |  | Breed | Boxer | | | AM | |  |  | | AM-colony | | MAS | | Siberian Husky | |
| --- | --- | --- | --- | --- | --- | --- | --- | --- | --- | --- | --- | --- | --- | --- | --- | --- | --- |
|  |  |  | *cd*- status | Normal | | | Carrier | | Affected | | | |  |  |  | Carrier | |
|  |  |  |  | N | | N | N | A | A | | A | A | A | A | A | A | N |
| # | Location on CFA29 | Distance from deletion in bp | Alleles |  | |  |  |  |  | |  |  |  |  |  |  |  |
| 1 | 32,670,916 | 3,028,462 | C/T | C | | T | T | C | C | | C | C | C | T | T | T | C |
| 2 | 32,671,054 | 3,028,324 | A/G | A | | G | G | A | A | | A | G | G | G | G | G | A |
| 3 | 32,671,176 | 3,028,202 | A/C | A | | C | A | A | A | | A | A | A | A | A | A | A |
| 4 | 33,671,259 | 2,028,119 | SINE | - | | - | - | - | - | | - | + | + | + | + | + | + |
| 5 | 33,671,277 | 2,028,101 | A/G |  | |  | A | A | A | | A | G | G | G | G | G | G |
| 6 | 33,671,337 | 2,028,041 | A/G |  | |  | G | G | G | | G | A | A | A | A | A | A |
| 7 | 33,671,933 | 2,027,445 | C/T | C | | C | C | C | C | | C | C | C | C | C | C | C |
| 8 | 33,672,100 | 2,027,278 | A/G | A | | A | A | A | A | | A | G | G | G | G | G | G |
| 9 | 33,672,109 | 2,027,269 | A/T | A | | T | A | A | A | | A | T | T | T | T | T | T |
| 10 | 33,672,169 | 2,027,209 | C/T | C | | T | T | T | T | | T | C | C | C | C | C | C |
| 11 | 33,672,300 | 2,027,078 | C/T | C | | T | C | C | C | | C | T | T | T | T | T | T |
| 12 | 33,672,320 | 2,027,058 | A/G | A | | G | A | A | A | | A | G | G | G | G | G | G |
| 13 | 33,672,329 | 2,027,049 | G/T | G | | T | T | T | T | | T | G | G | G | G | G | G |
| 14 | 33,672,382 | 2,026,996 | A/T | A | | T | T | T | T | | T | A | A | A | A | A | A |
| 15 | 33,672,420 | 2,026,958 | A/G | A | | G | A | A | A | | A | G | G | G | G | G | G |
| 16 | 33,672,487 | 2,026,891 | A/G | A | | G | A | A | A | | A | G | G | G | G | G | G |
| 17 | 33,672,490 | 2,026,888 | G/C | G | | C | C | C | C | | C | G | G | G | G | G | G |
| 18 | 33,672,522 | 2,026,856 | T/G | T | | G | T | T | T | | T | G | G | G | G | G | G |
| 19 | 33,672,549 | 2,026,829 | T/C | C | | C | C | C | C | | C | T | T | T | T | T | T |
| 20 | 33,672,641 | 2,026,737 | T/G | G | | G | G | G | G | | G | T | T | T | T | T | T |
| 21 | 33,672,715 | 2,026,663 | A/G | G | | G | G | G | G | | G | G | G | G | G | G | G |
| 22 | 33,672,779 | 2,026,599 | T/C | T | | T | T | T | T | | T | C | C | C | C | C | C |
| 23 | 33,672,903 | 2,026,475 | T/C | T | | C | T | C | C | | C | T | T | T | T | T | T |
| 24 | 34,173,985 | 1,525,393 | A/G | A | | A | A | A | A | | A | A | A | A | A | A | G |
| 25 | 34,174,010 | 1,525,368 | A/C | A | | A | A | A | A | | A | A | A | A | A | A | A |
| 26 | 34,174,058 | 1,525,320 | C/T | C | | C | C | C | C | | C | C | C | C | C | C | C |
| 27 | 34,174,073 | 1,525,305 | A/G | G | | G | G | G | G | | G | G | G | G | G | G | G |
| 28 | 34,174,286 | 1,525,092 | A/G | A | | A | A | A | A | | A | G | G | G | G | G | G |
| 29 | 34,162,404 | 1,536,974 | A/G | A | | A | G | G | G | | G | A | A | A | A | A | A |
| 30 | 34,162,664 | 1,536,714 | A/G | A | | A | G | G | G | | G | A | A | A | A | A | A |
| 31 | 34,163,059 | 1,536,319 | A/G | G | | G | A | A | A | | A | G | G | G | G | G | G |
| 32 | 34,163,082 | 1,536,296 | A/G | A | | A | G | G | G | | G | G | G | G | G | G | G |
| 33 | 34,163,140 | 1,536,238 | A/T | A | | A | T | T | T | | T | T | T | T | T | T | T |
| 34 | 34,163,155-8 | 1,536,220 | wt/del (ctgt) | wt | | wt | del | del | del | | del | del | del | del | del | del | del |
| 35 | 34,163,175 | 1,536,203 | T/C | T | | T | C | C | C | | C | C | C | C | C | C | C |
| 36 | 34,163,190 | 1,536,188 | T/A | T | | T | A | A | A | | A | T | T | T | T | T | T |
| 37 | 34,163,205 | 1,536,173 | A/G | G | | G | A | A | A | | A | A | A | A | A | A | A |
| 38 | 34,163,207 | 1,536,171 | T/A | A | | A | T | T | T | | T | T | T | T | T | T | T |
| 39 | 34,163,313 | 1,536,065 | C/T | C | | C | T | T | T | | T | C | C | C | C | C | C |
| 40 | 34,654,195 | 1,045,183 | A/C | A | | A | C | A | A | | A | C | C | C | C | C | A |
| 41 | 34,654,213 | 1,045,165 | T/C | T | | T | C | T | T | | T | C | C | C | C | C | T |
| 42 | 34,654,250 | 1,045,128 | A/G | G | | G | A | G | G | | G | A | A | A | A | A | G |
| 43 | 34,654,480 | 1,044,898 | A/G | G | | G | A | A | A | | A | A | A | A | A | A | A |
| 44 | 34,654,680 | 1,044,698 | C/T | C | | C | T | C | C | | C | T | T | T | T | T | C |
| 45 | 34,654,735 | 1,044,643 | C/T | C | | C | T | C | C | | C | T | T | T | T | T | C |
| 46 | 34,654,764 | 1,044,614 | A/C | A | | A | C | A | A | | A | C | C | C | C | C | A |
| 47 | 34,654,933 | 1,044,445 | T/C | T | | T | C | T | T | | T | C | C | C | C | C | T |
| 48 | 34,654,935 | 1,044,443 | A/G | A | | A | G | A | A | | A | G | G | G | G | G | A |
| 49 | 34,654,988 | 1,044,390 | A/G | G | | G | G | A | A | | A | G | G | G | G | G | A |
| 50 | 34,655,012 | 1,044,366 | A/C | A | | A | C | A | A | | A | C | C | C | C | C | A |
| 51 | 34,655,058 | 1,044,320 | C/T | C | | C | T | C | C | | C | T | T | T | T | T | C |
| 52 | 35,192,980 | 506,398 | C/T | C | | T | T | T | T | | T | T | T | T | T | T | T |
| 53 | 35,193,115 | 506,263 | G/T | G | | T | G | G | G | | G | G | G | G | G | G | G |
| 54 | 35,193,231 | 506,147 | C/T | T | | T | C | C | C | | C | C | C | C | C | C | T |
| 55 | 35,195,966 | 503,412 | C/A | A | | A | C | C | C | | C | C | C | C | C | C | A |
| 56 | 35,195,973 | 503,405 | C/T | C | | T | T | T | T | | T | T | T | T | T | T | C |
| 57 | 35,195,984 | 503,394 | C/T | C | | T | T | T | T | | T | T | T | T | T | T | C |
| 58 | 35,196,004 | 503,374 | C/T | C | | C | C | C | C | | C | C | C | C | C | C | T |
| 59 | 35,196,016 | 503,362 | C/T | C | | C | T | T | T | | T | T | T | T | T | T | C |
| 60 | 35,196,069 | 503,309 | C/A | C | | A | C | C | C | | C | C | C | C | C | C | C |
| 61 | 35,196,082 | 503,296 | C/T | C | | T | T | T | T | | T | T | T | T | T | T | C |
| 62 | 35,196,222 | 503,156 | C/T | C | | T | C | C | C | | C | C | C | C | C | C | T |
| 63 | 35,196,299 | 503,079 | C/T | C | | T | C | C | C | | C | C | C | C | C | C | T |
| 64 | 35,196,303 | 503,075 | A/C | A | | C | A | A | A | | A | A | A | A | A | A | C |
| 65 | 35,491,811 | 207,567 | T/C | T | | T | C | C | C | | C | C | C | C | C | C | T |
| 66 | 35,491,812 | 207,566 | G/A | G | | G | A | G | G | | G | G | G | G | G | G | A |
| 67 | 35,492,139 | 207,239 | T/C | T | | T | C | C | C | | C | C | C | C | C | C | C |
| 68 | 35,524,267 | 175,111 | G/C | G | | G | C | C | C | | C | C | C | C | C | C | G |
| 69 | 35,524,560 | 174,818 | A/G | A | | G | G | G | G | | G | G | G | G | G | G | G |
| 70 | 35,528,213 | 171,165 | C/T | C | | T | C | C | C | | C | C | C | C | C | C | C |
| 71 | 35,589,027 | 110,351 | A/T | A | | A | T | T | T | | T | T | T | T | T | T | A |
| 72 | 35,589,155 | 110,223 | A/T | A | | T | A | A | A | | A | A | A | A | A | A | A |
| 73 | 35,589,324 | 110,054 | A/G | A | | A | G | G | G | | G | G | G | G | G | G | A |
| 74 | 35,610,718 | 88,660 | C/T | T | | T | C | C | C | | C | C | C | C | C | C | T |
| 75 | 35,610,848 | 88,530 | G/T | G | | T | G | G | G | | G | G | G | G | G | G | G |
| 76 | 35,613,186 | 86,192 | A/G | G | | G | G | G | G | | G | G | G | G | G | G | G |
| 77 | 35,613,217 | 86,161 | GT repeat | 11 | | 11 | 20 | 20 | 20 | | 20 | 21 | 21 | 20 | 20 |  | 11 |
| 78 | 35,632,106 | 67,272 | T/G | G | | G | T | T | T | | T | T | T | T | T | T | G |
| 79 | 35,632,168 | 67,210 | T/C | T | | T | C | C | C | | C | C | C | C | C | C | T |
| 80 | 35,632,223 | 67,155 | A/T | T | | T | A | A | A | | A | A | A | A | A | A | T |
| 81 | 35,632,256 | 67,122 | C/T | C | | T | T | T | T | | T | T | T | T | T | T | C |
| 82 | 35,634,599 | 64,779 | A/C | A | | A | A | A | A | | A | A | A | A | A | A | A |
| 83 | 35,634,638 | 64,740 | C/T | C | | C | C | C | C | | C | C | C | C | C | C | C |
| 84 | 35,661,781 | 37,597 | A/G | A | | G | A | A | A | | A | A | A | A | A | A | G |
| 85 | 35,693,838 | 5,540 | A/T | A | | A | T | T | T | | T | T | T | T | T | T | T |
| 86 | 35,693,894 | 5,484 | A/G | A | | A | C | C | C | | C | C | C | C | C | C | C |
| 87 | 35,693,909 | 5,469 | A/G | G | | G | A | A | A | | A | A | A | A | A | A | G |
| 88 | 35,693,911 | 5,467 | A/G | A | | A | G | G | G | | G | G | G | G | G | G | G |
| 89 | 35,693,929 | 5,449 | G/T | T | | T | del | del | del | | del | del | del | del | del | del | T |
| 90 | 35,694,016 | 5,362 | C/T | C | | C | C | C | C | | C | C | C | C | C | C | T |
| 91 | 35,694,052 | 5,326 | G/T | G | | G | T | T | T | | T | T | T | T | T | T | G |
| 92 | 35,694,196 | 5,182 | G/C | C | | C | G | G | G | | G | G | G | G | G | G | C |
| 93 | 35,694,260 | 5,118 | A/G | G | | G | A | A | A | | A | A | A | A | A | A | G |
| 94 | 35,698,465 | 913 | G/A | A | | A | G | G | G | | G | G | G | G | G | G | G |
| 95 | 35,698,634 | 744 | G/C | G | | G | C | C | C | | C | C | C | C | C | C | C |
| 96 | 35,698,828 | 550 | C/T | C | | C | C | C | C | | C | C | C | C | C | C | T |
| 97 | 35,698,982 | 396 | A/C | C | | C | A | A | A | | A | A | A | A | A | A | A |
| 98 | 35,699,124 | 254 | C/T | C | | C | T | T | T | | T | T | T | T | T | T | T |
|  | 35,699,378-36,104,197 | 0 | 404,820 bases deletion | wt | | wt | wt | **mut** | **mut** | | **mut** | **mut** | **mut** | **mut** | **mut** | **mut** | wt |
| 99 | 36,104,213 | 16 | G/A | A | | A | G | G | G | | G | G | G | G | G | G | A |
| 100 | 36,104,546 | 349 | G/T | G | | G | T | T | T | | T | T | T | T | T | T | G |
| 101 | 36,104,725 | 528 | C/T | C | | T | C | C | C | | C | C | C | C | C | C | C |
| 102 | 36,105,137 | 940 | A/G | A | | G | G | G | G | | G | G | G | G | G | G | G |
| 103 | 36,105,138-41 | 941-944 | TCAG del | wt | | wt | del | del | del | | del | del | del | del | del | del | wt |
| 104 | 36,106,334 | 2,137 | G/A | A | | G | G | G | G | | G | G | G | G | G | G | G |
| 105 | 36,106,350 | 2,153 | A/G | G | | G | A | A | A | | A | A | A | A | A | G | G |
| 106 | 36,106,365 | 2,168 | T/C | C | | T | T | T | T | | T | T | T | T | T | T | T |
| 107 | 36,106,487 | 2,290 | A/G | A | | G | G | G | G | | G | G | G | G | G | A | A |
| 108 | 36,106,526 | 2,329 | A/T | T | | T | T | T | T | | T | T | T | T | T | T | T |
| 109 | 36,106,586 | 2,389 | C/G | C | | G | C | C | C | | C | C | C | C | C | G | G |
| 110 | 36,106,948 | 2,751 | A/G | G | | G | A | A | A | | A | A | A | A | A | A | G |
| 111 | 36,107,071 | 2,874 | C/T | C | | T | T | T | T | | T | T | T | T | T | T | C |
| 112 | 36,107,199 | 3,002 | C/T | T | | T | C | C | C | | C | C | C | C | C | C | T |
| 113 | 36,111,465 | 7,268 | A/G | G | | G | A | A | A | | A |  |  |  |  | A | G |
| 114 | 36,111,575 | 7,378 | C/T | T | | T | C | C | C | | C |  |  | C | C | C | T |
| 115 | 36,111,685 | 7,488 | A/G | G | | A | A | A | A | | A |  |  |  |  | A | A |
| 116 | 36,111,794 | 7,597 | A/G | G | | G | A | A | A | | A |  |  |  |  | A | G |
| 117 | 36,117,443 | 13,246 | A/G | A | | G | G | G | G | | G | G | G | G | G | G | A |
| 118 | 36,117,922 | 13,725 | A/G | A | | G | G | G | G | | G | G | G | G | G | G | A |
| 119 | 36,153,720 | 49,523 | C/T | T | | T | C | C | C | | C | C | C | C | C | C | T |
| 120 | 36,174,807 | 70,610 | A/G | A | | G | G | G | G | | G | G | G | G | G | G | G |
| 121 | 36,175,147 | 70,950 | A/G | A | | A | G | G | G | | G | G | G | G | G | G | A |
| 122 | 36,193,777 | 89,580 | A/C | A | | A | A | A | A | | A | A | A | A | A | A | A |
| 123 | 36,217,210 | 113,013 | A/G | A | | A | A | A | A | | A | A | A | A | A | A | A |
| 124 | 36,218,510 | 114,313 | C/T | C | | T | T | T | T | | T | T | T | T | T | T | T |
| 125 | 36,294,101 | 189,904 | A/G | A | | G | A | A | A | | A | A | A | A | A | A | A |
| 126 | 36,303,197 | 199,000 | G/T | G | | T | G | G | G | | G | G | G | G | G | G | G |
| 127 | 36,303,270 | 199,073 | T/A | A | | T | T | T | T | | T | T | T | T | T | T | T |
| 128 | 36,304,349 | 200,152 | G/T | T | | T | T | T | T | | T | T | T | T | T | T | T |
| 129 | 36,304,350 | 200,153 | G/T | T | | T | T | T | T | | T | T | T | T | T | T | T |
| 130 | 36,304,363 | 200,166 | A/G | A | | A | A | A | A | | A | A | A | A | A | A | A |
| 131 | 36,304,375 | 200,178 | A/C | A | | A | A | A | A | | A | A | A | A | A | A | A |
| 132 | 36,304,435 | 200,238 | A/C | C | | C | C | C | C | | C | C | C | C | C | C | C |
| 133 | 36,304,566 | 200,369 | T/C | T | | T | T | T | T | | T | T | T | T | T | T | T |
| 134 | 36,304,692 | 200,495 | T/G | T | | T | T | T | T | | T | T | T | T | T | T | T |
| 135 | 36,304,796 | 200,599 | A/G | G | | G | G | G | G | | G | G | G | G | G | G | G |
| 136 | 36,304,808 | 200,611 | A/G | G | | G | G | G | G | | G | G | G | G | G | G | G |
| 137 | 36,304,845 | 200,648 | A/C | A | | A | A | A | A | | A | A | A | A | A | A | A |
| 138 | 36,604,194 | 499,997 | Tetra repeat | Het | | | Het. | | 10 | | 10 | 10 | 10 | 9 | 9 | 10 | 11 |
| 139 | 36,604,320 | 500,123 | A/G | A | A | |  |  | A | | A | A | A | A | A | A | A |
| 140 | 36,604,369 | 500,172 | A/T | T | T | |  |  | T | | T | T | T | T | T | T | T |
| 141 | 36,604,540 | 500,343 | T/C | T | T | |  |  | T | | T | T | T | T | T | T | T |
| 142 | 36,604,565 | 500,368 | T/C | T | T | |  |  | C | | C | C | C | C | C | C | T |
| 143 | 36,604,709 | 500,512 | A/T | A | A | |  |  | A | | A | A | A | A | A | A | A |
| 144 | 36,604,913 | 500,716 | A/G | A | G | | G | G | G | | G | G | G | G | G | G | A |
| 145 | 36,605,094 | 500,897 | A/G | G | G | | G | G | G | | G | G | G | G | G | G | A |
| 146 | 36,605,212 | 501,015 | G/T | G | G | | G | G | G | | G | G | G | G | G | G | G |
| 147 | 36,605,436 | 501,239 | C/T | C | T | | C | T | T | | T | T | T | T | T | T | T |
